# Modelling the Formation of Liver Zones within the Scope of Fractional Order Derivative

**DOI:** 10.1155/2014/478028

**Published:** 2014-09-08

**Authors:** Abdon Atangana, Suares Clovis Oukouomi Noutchie

**Affiliations:** ^1^Institute for Groundwater Studies, Faculty of Natural and Agricultural Sciences, University of the Free State, Bloemfontein 9301, South Africa; ^2^MaSIM Focus Area, North-West University, Mafikeng 2735, South Africa

## Abstract

We develop and extend earlier results related to mathematical modelling of the liver formation zone by the adoption of noninteger order derivative. The hidden uncertainties in the model are captured and controlled thanks to the Caputo derivative. The stationary states are investigated and the time-dependent solution is approximated using two recent iteration methods. In particular, we discuss the convergence of these methods by constructing a suitable Hilbert space.

## 1. Introduction

One of the most important parts of any animal or human being body is the liver. The liver is a very important organ of vertebrates and many other flora and fauna [1]. Its properties include counting detoxification, protein synthesis, and production of biochemicals indispensable for absorption [2]. The liver is indispensable for continued existence; there is at this time no way to pay damages for the nonexistence of liver meaning in the long term, even though novel liver dialysis practices can be used in the temporary [1, 2]. This gland shows business of a main role in metabolism and has a number of functions in the body, including glycogen storage, decomposition of red blood cells, plasma protein synthesis, hormone production, and detoxification [3]. It is found below the diaphragm in the abdominal-pelvic area of the abdomen. It brings into being bile, an alkaline compound which aids in digestion via the emulsification of lipids. The liver's extremely dedicated tissues normalize an extensive diversity of high-volume biochemical reactions, including the synthesis and breakdown of small and complex molecules, countless of which are essential for ordinary fundamental functions [3].

**Figure 1:**
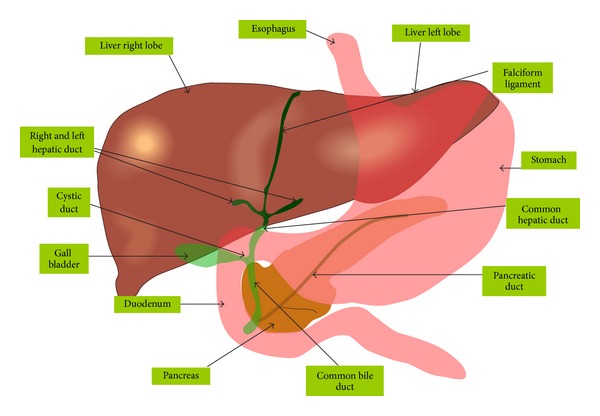
Anatomy of the biliary tree, liver, and gall bladder (From Jiju Kurian Punnoose, 10/12/2007).

The human liver is typically separated into two lobes (left and right), if viewed from the parietal surface, but if observed on the visceral surface it is divided into four lobes with the addition of the caudate and quadrate lobe [3]. Other anatomical landmarks exist, such as the ligamentum venosum (ligamentum of Arancio) and the round ligament (ligamentum Teres) that further divide the left side of the liver in two sections [4]. The falciform ligament is visible on the front (anterior side) of the liver. This divides the liver into a left anatomical lobe and a right anatomical lobe [1–4]. Two most important types of cells inhabit the liver lobes: karat parenchymal and nonparenchymal cells. 80% of the liver volume is engaged by parenchymal cells normally referred to as hepatocytes. Nonparenchymal cells comprise 40% of the total number of liver cells but only 6.5% of its volume [3]. Sinusoidal hepatic endothelial cells, Kupffer cells, and hepatic stellate cells are some of the nonparenchymal cells that line the liver sinusoid [3]. A schematic representation of anatomy of the biliary tree, liver, and gall bladder is shown in Figure 1.

More than a few metabolic occupations of the liver have been established to be prearranged in spatial zones arranged in relation to the direction of hepatic blood flow, in such a way that a quantity of enzymes operate approximately together upstream others. A group of the most eminent scholars who studied this phenomenon are Bass et al. [5]. In one of their investigations, they qualified such distributions of enzymes activities to distributions of cell types [5]. For the simplest case of two enzymes, there are two corresponding cell types, each containing only one of the enzymes; separate metabolic zones occur when all cells of one type are located upstream all cells of the other type. Additionally, it was stated in [5] that each cell type reproduces itself by division.

The mathematical model of the formation of liver zone was discussed in [5]. However in order to accommodate readers that are not in the field of mathematical medicine, we will discuss for expediency the main steps in its derivation in the next section.

In the recent decades it was revealed by several scholars dealing with real world problems that the models using the concept of noninteger order derivatives are more suitable in prediction than those with ordinary derivatives. One of the goals of this paper is the analysis of the model within the folder of fractional calculus including the steady state analysis, a possible analytical solution using the recent development of analytical methods.

## 2. Mathematical Formulation

The mathematical model describing the formation of liver zones is a system of nonlinear integropartial differential equations. Approximately 1100 mL of blood flows from the portal vein into the liver sinusoids each minute, and approximately an additional 350 mL flows into the sinusoids from the hepatic artery, and the total averaging is about 1450 mL/min. This amounts to about 29% of the resting cardiac output [6]. As the many capillaries comprising the liver are similar and act essentially in parallel, Bass et al. [5] modelled a representative capillary lined with cells of two kinds. In the investigation done by Bass et al, it was recommended to put the *x*-axis along the blood flow, with bay at *x* = 0 and outlet at *x* = *L*. The density of cells of the first and second kinds is defined by *ρ*_1_(*x*, *t*) and *ρ*_2_(*x*, *t*), respectively, as a continuous representation of the number of cells of the first kind and second per unit length of capillary at time *t* at the position *x*. It was suggested in their investigation that the total cell density *ρ*_1_ + *ρ*_2_ cannot go beyond a fixed highest density *σ* of cell sites, as division of the cell is limited by the well-known phenomenon of contact inhibition. The mathematical formulation describing the above phenomena is given as follows:

(1) $$\begin{array}{c} \frac{\partial \rho_{1}}{\partial t}=K_{1}\rho_{1}\left(\sigma -\rho_{1}-\rho_{2}\right)-\beta_{1}\left(c\right)\rho_{1},\\ \frac{\partial \rho_{2}}{\partial t}=K_{2}\rho_{2}\left(\sigma -\rho_{1}-\rho_{2}\right)-\beta_{2}\left(c\right)\rho_{2},\\ c=c_{0}-\frac{1}{f}\overset{x}{\underset{0}{\int }}\left[k_{1}\rho_{1}+k_{2}\rho_{2}\right]d\varphi . \end{array}$$

Here *k*_1_ and *k*_2_ are positive constants and *c* is the concentration of a controlling blood-borne substance. The above system was reduced in [5] as follows:

(2) $$\begin{array}{c} \frac{\partial \rho_{1}}{\partial t}=K_{1}\rho_{1}\left(\sigma -\rho_{1}-\rho_{2}\right)-\mu_{1}-\rho_{1}\frac{\gamma_{1}}{f}\overset{x}{\underset{0}{\int }}\left[k_{1}\rho_{1}+k_{2}\rho_{2}\right]d\varphi ,\\ \frac{\partial \rho_{2}}{\partial t}=K_{2}\rho_{2}\left(\sigma -\rho_{1}-\rho_{2}\right)-\mu_{2}-\rho_{2}\frac{\gamma_{2}}{f}\overset{x}{\underset{0}{\int }}\left[k_{1}\rho_{1}+k_{2}\rho_{2}\right]d\varphi . \end{array}$$

In this paper we are not going to consider the above system since in recent decades it was revealed by several scholars dealing with real world problems that the models using the concept of noninteger order derivatives are more suitable in prediction than those with ordinary derivative [7–10]. In order to take into account all uncertainties that can be associated with the formation of the liver zone in human or animal body, we will convert the ordinary derivative to the concept of noninteger order derivative as follows:

(3) ∂αρ1∂tα=[K1(σ−ρ1−ρ2)−μ1  −γ1fΓ(α)∫0x(x−φ)α−1[k1ρ1+k2ρ2]dφ]ρ1,∂αρ2∂tα=[K2(σ−ρ1−ρ2)−μ2  −γ2fΓ(α)∫0x(x−φ)α−1[k1ρ1+k2ρ2]dφ]ρ2,

where 0 < *α* ≤ 1, *μ*_*i*_ *i* = 1,2, are death rate of kinds 1 and 2, respectively, and *γ*_*i*_ *i* = 1,2 are constants. This conversion also implies that the global concentration of a controlling blood-borne substance is presented as

(4) $$\begin{array}{c} c\left(x,t,\alpha \right)=c_{0}-\frac{1}{f\Gamma \left(\alpha \right)}\overset{x}{\underset{0}{\int }}{\left(x-\varphi \right)}^{\alpha -1}\left[k_{1}\rho_{1}+k_{2}\rho_{2}\right]d\varphi , \end{array}$$

where Γ is the gamma function defined as

(5) $$\begin{array}{c} \Gamma \left(x\right)=\overset{\infty }{\underset{0}{\int }}t^{x-1}e^{-t}dt. \end{array}$$

It is worth noting that if *α* = 1, we revert the conventional model of liver zone formation. The fractional derivative used here is in Caputo sense. There exists more than one definition of fractional derivative in the literature, and we will list the most common ones. The most popular ones are the Riemann-Liouville and the Caputo derivatives [8–10]. For Caputo derivative we have

(6) D0Cxα(f(x))=1Γ(n−α)∫0x(x−t)n−α−1dnf(t)dtndt.

For the case of Riemann-Liouville we have the following definition:

(7) $$\begin{array}{c} D_{x}^{\alpha }\left(f\left(x\right)\right)=\frac{1}{\Gamma \left(n-\alpha \right)}\frac{d^{n}}{dx^{n}}\overset{x}{\underset{0}{\int }}{\left(x-t\right)}^{n-\alpha -1}f\left(t\right)dt. \end{array}$$

It was shown in [7–9] that the Caputo type is suitable for modelling real world problem, and based on this we will use throughout this paper the Caputo derivative.

## 3. Steady State Analysis

This section is devoted to the discussions concerning the stationary solutions, which means that we will consider that the densities are not depending on time. One of the enjoyable properties of the Caputo fractional derivative is that the fractional derivative of a constant is zero. Now based on that property, we assume that the densities of first and second kind do not depend on time; (3) will be reduced to

(8) 0=[K1(σ−ρ1∗−ρ2∗)−μ1  −γ1fΓ(α)∫0x(x−φ)α−1[k1ρ1∗+k2ρ2∗]dφ]ρ1,0=[K2(σ−ρ1∗−ρ2∗)−μ2  −γ2fΓ(α)∫0x(x−φ)α−1[k1ρ1∗+k2ρ2∗]dφ]ρ2.

This simply implies

(9) K1(σ−ρ1∗−ρ2∗)−μ1 −γ1fΓ(α)∫0x(x−φ)α−1[k1ρ1∗+k2ρ2∗]dφ with  ρ2=0,

(10a) K2(σ−ρ1∗−ρ2∗)−μ2 −γ2fΓ(α)∫0x(x−φ)α−1[k1ρ1∗+k2ρ2∗]dφ, with  ρ1=0,

(10b) $$\begin{array}{c} \rho_{1}-\rho_{2}=0, \end{array}$$

(11) K1(σ−ρ1∗−ρ2∗)−μ1 −γ1fΓ(α)∫0x(x−φ)α−1[k1ρ1∗+k2ρ2∗]dφ with  ρ2∗≠0,K2(σ−ρ1∗−ρ2∗)−μ2 −γ2fΓ(α)∫0x(x−φ)α−1[k1ρ1∗+k2ρ2∗]dφ, with  ρ1∗≠0.

We will start with the stationary solution of system (9). We will employ two different analytical techniques to get to the bottom of the system. We will start with the Laplace transform method:

(12) $$\begin{array}{c} K_{1}\left(\sigma -\rho_{1}^{*}\right)-\mu_{1}-\frac{\gamma_{1}}{f\Gamma \left(\alpha \right)}\overset{x}{\underset{0}{\int }}{\left(x-\varphi \right)}^{\alpha -1}\left[k_{1}\rho_{1}^{*}\right]d\varphi =0. \end{array}$$

Now if we put for simplicity *d*_1_ = *σ* − (*μ*_1_/*K*_1_), *a*_1_ = *k*_1_ *γ*_1_/*fK*_1_ with rearranging we obtain the following:

(13) $$\begin{array}{c} \rho_{1}^{*}=d_{1}-a_{1}\frac{1}{\Gamma \left(\alpha \right)}\overset{x}{\underset{0}{\int }}{\left(x-\varphi \right)}^{\alpha -1}\left[\rho_{1}^{*}\right]d\varphi . \end{array}$$

We will first show the properties of the Laplace transform in fractional calculus [8–10].

The Laplace transform of the function *f* is defined as follows:

(14) $$\begin{array}{c} L\left(f\left(x\right)\right)\left(s\right)=\overset{\infty }{\underset{0}{\int }}e^{-sx}f\left(x\right)dx. \end{array}$$

Let us observe the Laplace transform of the fractional derivative with Caputo

(15) L(D0Ctαf(x))(s)=sαF(s)−∑k=0n−1sα−k−1f(k)(0),              (n−1<α≤n).

The above uses the usual initial conditions or values of the functions. Also the Laplace transform of fractional integral is given as

(16) L(Jtαf(x))(s)=s−αF(s),  with  Jαf(x)=1Γ(α)∫0x(x−t)α−1f(t)dt,           α>0, x>0.

*F* is the Laplace transform of *f* and *s* the Laplace variable. Therefore applying the Laplace transform on both sides of (12), we arrive at

(17) $$\begin{array}{c} L\left\{\rho_{1}^{*}\left(x\right)\right\}\left(s\right)=\frac{d_{1}}{s+a_{1}s^{1-\alpha }}. \end{array}$$

Now applying the inverse-Laplace transform operator on both sides of (17) we arrive at

(18) $$\begin{array}{c} \rho_{1}^{*}\left(x\right)=L^{-1}\left[\frac{d_{1}}{s+a_{1}s^{1-\alpha }}\right]\left(x\right)=d_{1}E_{\alpha }\left[-{\left({a_{1}}^{1/\alpha }x\right)}^{\alpha }\right], \end{array}$$

where *E*_*α*_ is the so-called Mittag-Leffler function defined as

(19) $$\begin{array}{c} E_{\alpha }\left(x\right)=\overset{\infty }{\underset{n=0}{\sum }}\frac{x^{n}}{\Gamma \left(\alpha n+1\right)}. \end{array}$$

The above solution can be retrieved by using new recent development of iteration methods. This method is being proposed to avoid the stability of Laplace transform method on one hand. Also the Laplace transform method is only useful in the case of linear equation; however, the second method is used for both linear and nonlinear equations.

In this method we assume that the solution of (13) is in the form of

(20) $$\begin{array}{c} \rho_{1}^{*}\left(x\right)=\underset{p\to 1}{\lim}\overset{\infty }{\underset{n=0}{\sum }}p^{n}\rho_{1n}\left(x\right). \end{array}$$

Now substituting the above in (13) we obtain

(21) $$\begin{array}{c} \overset{\infty }{\underset{n=0}{\sum }}p^{n}\rho_{1n}\left(x\right)=d_{1}-a_{1}\frac{p}{\Gamma \left(\alpha \right)}\overset{x}{\underset{0}{\int }}{\left(x-\varphi \right)}^{\alpha -1}\left[\overset{\infty }{\underset{n=0}{\sum }}p^{n}\rho_{1n}\left(x\right)\right]d\varphi . \end{array}$$

Comparing terms of the same power of *p* yields the following iteration:

(22)  ρ10= d1, ρ11(x)=−a1Γ(α)∫0x(x−φ)α−1[ρ10(φ)]dφ, ρ12(x)=−a1Γ(α)∫0x(x−φ)α−1[ρ11(φ)]dφ, ρ1n(x)=−a1Γ(α)∫0x(x−φ)α−1[ρ1(n−1)(φ)]dφ.

Integrating the above set of integral equations, we arrive at the following result:

(23)  ρ10= d1, ρ11(x)=−d1a1xαΓ(α+1), ρ12(x)= d1(−a1)2(x)2αΓ(2α+1), ρ1n(x)= d1(−a1)n(x)nαΓ(nα+1).

Implying the stationary solution of density of the first kind is given as

(24) $$\begin{array}{c} \rho_{1}^{*}\left(x\right)=d_{1}\overset{\infty }{\underset{n=0}{\sum }}\frac{{\left({-a}_{1}\right)}^{n}{\left(x\right)}^{n\alpha }}{\Gamma \left(n\alpha +1\right)}=d_{1}E_{\alpha }\left[-{\left({\left(a_{1}\right)}^{1/\alpha }x\right)}^{\alpha }\right]. \end{array}$$

It was argued in the work done in [5] that there exists a certain point *x* = x* above which the density of the second kind *ρ*_2_(*x*) will approach a stationary, which can be determined by solving (25). Therefore in that interval we have the following equation:

(25) K2(σ−ρ2∗)−μ2−γ2fΓ(α)∫0x∗(x−φ)α−1[k1ρ1∗]dφ  −γ2fΓ(α)∫x∗x(x−φ)α−1[k2ρ2∗]dφ=0.

We can rearrange the above equation as follows:

(26) $$\begin{array}{c} \rho_{2}^{*}\left(x\right)=F\left(x^{*}\right)-\frac{\gamma_{2}k_{2}}{fK_{2}\Gamma \left(\alpha \right)}\overset{x}{\underset{x^{*}}{\int }}{\left(x-\varphi \right)}^{\alpha -1}\left[\rho_{2}^{*}\right]d\varphi ,\\ F\left(x^{*}\right)=\sigma -\frac{\mu_{2}}{K_{2}}-\frac{\gamma_{2}}{f\Gamma \left(\alpha \right)K_{2}}\overset{x^{*}}{\underset{0}{\int }}{\left(x-\varphi \right)}^{\alpha -1}\left[k_{1}\rho_{1}^{*}\right]d\varphi . \end{array}$$

For simplicity, let us put

(27) $$\begin{array}{c} \frac{\gamma_{2}k_{2}}{fK_{2}}=a_{2},\\ \rho_{2}^{*}\left(x\right)=F\left(x^{*}\right)-\frac{a_{2}}{\Gamma \left(\alpha \right)}\overset{x}{\underset{x^{*}}{\int }}{\left(x-\varphi \right)}^{\alpha -1}\left[\rho_{2}^{*}\right]d\varphi . \end{array}$$

Using a similar method, we obtain

(28)  ρ20= F(x∗), ρ21(x)=−a2Γ(α)∫0x(x−φ)α−1[ρ20(φ)]dφ, ρ22(x)=−a2Γ(α)∫0x(x−φ)α−1[ρ21(φ)]dφ, ρ2n(x)=−a2Γ(α)∫0x(x−φ)α−1[ρ2(n−1)(φ)]dφ.

Integrating the above set of integral equations, we arrive at the following result:

(29)  ρ20= F(x∗) ρ21(x)=−F(x∗)a2(x−x∗)αΓ(α+1), ρ22(x)= F(x∗)(−a2)2(x−x∗)2αΓ(2α+1), ρ2n(x)= F(x∗)(−a2)n(x−x∗)nαΓ(nα+1).

Implying the stationary solution of density of the second kind is given as

(30) ρ1∗(x)=F(x∗)∑n=0∞(−a2)n(x−x∗)nαΓ(nα+1)=F(x∗)Eα[−((a2)1/α(x−x∗))α].

Our next concern is to provide the stationary solutions for the following system:

(31) K1(σ−ρ1∗−ρ2∗)−μ1 −γ1fΓ(α)∫0x(x−φ)α−1[k1ρ1∗+k2ρ2∗]dφ=0               with  ρ2∗≠0,K2(σ−ρ1∗−ρ2∗)−μ2 −γ2fΓ(α)∫0x(x−φ)α−1[k1ρ1∗+k2ρ2∗]dφ=0,               with  ρ1∗≠0.

Dividing the first equation of the system by *K*_1_ and the second by *K*_2_ and subtracting the result of the first from the result of the second, we obtain

(32) 1Γ(α)∫0x(x−φ)α−1[k1ρ1∗+k2ρ2∗]dφ =(μ1/K1)−(μ2/K2)(γ2/fK2)−(γ1/fK1).

Now replacing the above result in the system, we arrive at

(33) σ−ρ1∗−ρ2∗=μ1+γ1fK1(μ1/K1)−(μ2/K2)(γ2/fK2)−(γ1/fK1)=μ2+γ2fK2(μ1/K1)−(μ2/K2)(γ2/fK2)−(γ1/fK1).

A condition for the above to hold is that (*γ*_2_/*fK*_2_)−(*γ*_1_/*fK*_1_) ≠ 0, (  *μ*_1_/*K*_1_)−(*μ*_2_/*K*_2_) must have the same sign with (*γ*_2_/*fK*_2_)−(*γ*_1_/*fK*_1_), and the ultimate best is that

(34) $$\begin{array}{c} \frac{\mu_{1}}{K_{1}}>\frac{\mu_{2}}{K_{2}},\;\;\frac{\gamma_{2}}{fK_{2}}>\frac{\gamma_{1}}{fK_{1}}. \end{array}$$

Thus, it is confirmed in this analysis that, even with stationary solutions,

(35) $$\begin{array}{c} \sigma -\rho_{1}^{*}-\rho_{2}^{*}>0. \end{array}$$

We will now continue our investigation by presenting methods to find the solutions of system (3). This is therefore done in the next section.

## 4. Finding Approximate Global Solution

Many real world problems have been modelled via differential equations. It is important to point out that once these physical problems are converted into mathematical equation, their solutions are further used to predict the future behaviors of these physical problems. It is therefore very important to develop analytical methods in order to find the solutions. Some of these equations are very difficult to handle analytically due to their complexity especially in the case of fractional calculus. In the last years, many scientists focused their regards on setting up techniques that can be used to solve these equations. But for our purpose, we will use only two of these techniques to present an approximate solution of (3).

### 4.1. Iteration Method Using Laplace Transform

This method was proposed by Khan and Wu to avoid calculation of Adomian polynomial and the use of correction function of the variational iteration method; the reference of this work is in [11]. One can also find the methodology of this efficient method in there, but here we will use it only. Now applying the Laplace transform on both sides of the below system, we arrive at

(36) ∂αρ1∂tα=[K1(σ−ρ1−ρ2)−μ1  −γ1fΓ(α)∫0x(x−φ)α−1[k1ρ1+k2ρ2]dφ]ρ1,∂αρ2∂tα=[K2(σ−ρ1−ρ2)−μ2  −γ2fΓ(α)∫0x(x−φ)α−1[k1ρ1+k2ρ2]dφ]ρ2

and the following

(37) sαL[ρ1  (x,t)](s) =L[K1(σ−ρ1−ρ2)−μ1    −γ1fΓ(α)∫0x(x−φ)α−1[k1ρ1+k2ρ2]dφ]ρ1,sαL[ρ2(x,t)](s) =L[K2(σ−ρ1−ρ2)−μ2    − γ2fΓ(α)∫0x(x−φ)α−1[k1ρ1+k2ρ2]dφ]ρ2.

For simplicity, let us put

(38) $$\begin{array}{c} L\left[\rho_{1,2\;}\left(x,t\right)\right]\left(s\right)=\overset{ˇ}{\rho_{1,2}}. \end{array}$$

Now taking the inverse Laplace operator on both sides of (37), we arrive at

(39) ρ1  (x,t)=ρ1(x,0) +L−1[1sα[K1(σ−ρ1−ρ2)−μ1−γ1fΓ(α)      ×∫0x(x−φ)α−1[k1ρ1+k2ρ2]dφ]ρ1],ρ2  (x,t)=ρ2(x,0) +L−1[1sα[K2(σ−ρ1−ρ2)−μ2−γ2fΓ(α)      ×∫0x(x−φ)α−1[k1ρ1+k2ρ2]dφ]ρ2].

Since we are not yet sure of the exact solution of the above system, we will assume that the solution can be represented in series form according to the Poincare idea; that is,

(40) ρ1(x,t)=∑n=0∞pnρ1n(x,t),  ρ2(x,t)=∑n=0∞pnρ2n(x,t).

The above can be substituted in (39); however, comparing terms of the same power of *p* we arrived at the following set:

(41) ρ10(x,t)=ρ1  (x,0),    ρ20(x,t)=ρ2(x,0),ρ11(x,t) =+L−1[1sα[K1(σ−ρ10−ρ20)−μ1−γ1fΓ(α)       ×∫0x(x−φ)α−1[k1ρ10+k2ρ20]dφ]ρ10],ρ21(x,t) =+L−1[1sα[K1(σ−ρ10−ρ20)−μ2−γ2fΓ(α)      ×∫0x(x−φ)α−1[k1ρ10+k2ρ20]dφ]ρ20].

In general for *n* greater than 1 we can obtain the rest of the terms with the following recursive formula:

(42) ρ1,n+1(x,t)=L−1[1sαL[K1σρ1,n−K1∑j=0nρ1,n−j−1ρ1j      −K1∑j=0nρ2,n−j−1ρ1j−μ1ρ1,n−ρ1nJ1αn(x,t)]],ρ2,n+1(x,t)=L−1[1sαL[K2σρ2,n−K2∑j=0nρ1,n−j−1ρ1j      −K2∑j=0nρ2,n−j−1ρ1j−μ1ρ1,n−ρ1nJ2αn(x,t)]]

with of course

(43) $$\begin{array}{c} {J_{2\alpha }}^{n}\left(x,t\right)=\frac{\gamma_{2}}{f\Gamma \left(\alpha \right)}\overset{x}{\underset{0}{\int }}{\left(x-\varphi \right)}^{\alpha -1}\left[k_{1}\rho_{1n}+k_{2}\rho_{2n}\right]d\varphi ,\\ {J_{1\alpha }}^{n}\left(x,t\right)=\frac{\gamma_{1}}{f\Gamma \left(\alpha \right)}\overset{x}{\underset{0}{\int }}{\left(x-\varphi \right)}^{\alpha -1}\left[k_{1}\rho_{1n}+k_{2}\rho_{2n}\right]d\varphi . \end{array}$$

We will summarize the entire procedure in the following algorithm.

**Algorithm 1 .**

(i) Input: *I*_1_(*x*), *I*_2_(*x*)—as initial conditions, meaning in our case *ρ*_1_(*x*, 0) and *ρ*_1_(*x*, 0), *j*—number terms in the rough calculation.

(ii) Output: *ρ*_1approx_(*x*, *t*), *ρ*_2approx_(*x*, *t*) the approximate solutions.

**Step 1 .**

Put *ρ*_10_(*x*, *t*) = *I*_1_(*x*), *ρ*_20_(*x*, *t*) = *I*_2_(*x*) and *ρ*_1approx_(*x*, *t*) = *ρ*_10_(*x*, *t*), *ρ*_2approx_(*x*, *t*) = *I*_2_(*x*).

**Step 2 .**

For *j* = 0 to *n* − 1 do Step 3, Step 4, and Step 5.

**Step 3 .**

Compute

(44) M1n=K1σρ1,n−K1∑j=0nρ1,n−j−1ρ1j −K1∑j=0nρ2,n−j−1ρ1j−μ1ρ1,n−ρ1nJ1αn(x,t),M2n=K2σρ2,n−K2∑j=0nρ1,n−j−1ρ1j −K2∑j=0nρ2,n−j−1ρ1j−μ1ρ1,n−ρ1nJ2αn(x,t).

**Step 4 .**

Compute

(45) $$\begin{array}{c} \rho_{1,n+1}\left(x,t\right)=L^{-1}\left[\frac{1}{s^{\alpha }}L\left[M_{1n}\right]\right],\\ \rho_{2,n+1}\left(x,t\right)=L^{-1}\left[\frac{1}{s^{\alpha }}L\left[M_{2n}\right]\right]. \end{array}$$

**Step 5 .**

Compute *ρ*_1approx_(*x*, *t*) = *ρ*_1approx_(*x*, *t*) + *ρ*_1,*n*+1_(*x*, *t*), *ρ*_2approx_(*x*, *t*) = *ρ*_2approx_(*x*, *t*) + *ρ*_2,*n*+1_(*x*, *t*).

Stop.

### 4.2. Iteration Method Using the Integral Transform

This version of homotopy was first proposed by Abdon Atangana and used to solve the groundwater flow equation in the confined aquifer [12, 13]. This version uses the idea of integral transform coupling with the Poincare series that was previously proposed by He [14]. This version is easier to use as one can see in this case.

The first step in this approach is to apply the inverse operator of ∂^*α*^/∂*t* ^*α*^ on both sides of equation to obtain the following equation:

(46) ρ1=ρ1(x,0) +∫0t(t−φ)α−1Γ(α)  ×[K1(σ−ρ1−ρ2)−μ1−γ1fΓ(α)    ×∫0x(x−φ)α−1[k1ρ1+k2ρ2]dφ]ρ1dφ,ρ2=ρ2(x,0) +∫0t(t−φ)α−1Γ(α)  ×[K2(σ−ρ1−ρ2)−μ2−γ2fΓ(α)    ×∫0x(x−φ)α−1[k1ρ1+k2ρ2]dφ]ρ2dφ.

Since it is not certain that the above has exact solution, the second step of the method proposes that the solution should be in the series of Poincare meaning:

(47) $$\begin{array}{c} \rho_{1}\left(x,t\right)=\overset{\infty }{\underset{n=0}{\sum }}p^{n}\rho_{1n}\left(x,t\right),\;\;\rho_{2}\left(x,t\right)=\overset{\infty }{\underset{n=0}{\sum }}p^{n}\rho_{2n}\left(x,t\right). \end{array}$$

Then *ρ*_1,2_ can be included in (46) and after including the imbedding parameter *p* and comparing terms of the same power of *p* we arrive at the following set of equations:

(48) ρ10(x,t)=ρ1  (x,0),  ρ20(x,t)=ρ2(x,0),ρ11(x,t)=∫0t(t−φ)α−1Γ(α) ×[K1(σ−ρ10−ρ20)−μ1−γ1fΓ(α)   ×∫0x(x−φ)α−1[k1ρ10+k2ρ20]dφ]ρ10dψ,ρ21(x,t)=∫0t(t−φ)α−1Γ(α) ×[K1(σ−ρ10−ρ20)−μ2−γ2fΓ(α)   ×∫0x(x−φ)α−1[k1ρ10+k2ρ20]dφ]ρ20dψ.

In general for *n* greater than 1 we can obtain the rest of the terms with the following iteration formula:

(49) ρ1,n+1(x,t) =∫0t(t−φ)α−1Γ(α)  ×[K1σρ1,n−K1∑j=0nρ1,n−j−1ρ1j    −K1∑j=0nρ2,n−j−1ρ1j−μ1ρ1,n−ρ1nJ1αn(x,t)]dψ,ρ2,n+1(x,t) =∫0t(t−φ)α−1Γ(α)  ×[K2σρ2,n−K2∑j=0nρ1,n−j−1ρ1j    −K2∑j=0nρ2,n−j−1ρ1j−μ1ρ1,n−ρ1nJ2αn(x,φ)]dψ.

As soon as one is provided with initial condition, this algorithm can be implemented easily. To make this simple, we provide the summary of the procedure for this case in the following algorithm.

**Algorithm 2 .**

(i) Put in: *I*_1_(*x*), *I*_2_(*x*)—as preliminary conditions, meaning in our case *ρ*_1_(*x*, 0) and *ρ*_1_(*x*, 0), *k*—number terms in the rough calculation.

(ii) Output: *ρ*_1approx_(*x*, *t*), *ρ*_2approx_(*x*, *t*) the approximate solutions.

**Step 1 .**

Put *ρ*_10_(*x*, *t*) = *I*_1_(*x*), *ρ*_20_(*x*, *t*) = *I*_2_(*x*) and *ρ*_1approx_(*x*, *t*) = *ρ*_10_(*x*, *t*), *ρ*_2approx_(*x*, *t*) = *I*_2_(*x*).

**Step 2 .**

For *k* = 0 to *n* − 1 do Step 3, Step 4, and Step 5.

**Step 3 .**

Compute

(50) L1n=K1σρ1,n−K1∑j=0nρ1,n−j−1ρ1j −K1∑j=0nρ2,n−j−1ρ1j−μ1ρ1,n−ρ1nJ1αn(x,t),L2n=K2σρ2,n−K2∑j=0nρ1,n−j−1ρ1j −K2∑j=0nρ2,n−j−1ρ1j−μ1ρ1,n−ρ1nJ2αn(x,t).

**Step 4 .**

Compute

(51) $$\begin{array}{c} \rho_{1,n+1}\left(x,t\right)=\overset{t}{\underset{0}{\int }}\frac{{\left(t-\varphi \right)}^{\alpha -1}}{\Gamma \left(\alpha \right)}L_{1n}d\psi ,\\ \rho_{2,n+1}\left(x,t\right)=\overset{t}{\underset{0}{\int }}\frac{{\left(t-\varphi \right)}^{\alpha -1}}{\Gamma \left(\alpha \right)}L_{2n}d\psi . \end{array}$$

**Step 5 .**

Compute *ρ*_1approx_(*x*, *t*) = *ρ*_1approx_(*x*, *t*) + *ρ*_1,*n*+1_(*x*, *t*), *ρ*_2approx_(*x*, *t*) = *ρ*_2approx_(*x*, *t*) + *ρ*_2,*n*+1_(*x*, *t*).

Stop.

**Remark 3 .**

A direct comparison of both methods shows that they all use integral transform and the Poincare series. However in the first method, one will first apply the Laplace transform and again apply the inverse-Laplace operator which sometime can be very difficult to handle due to the instability of the Laplace transform operator. But the second method uses just a simple integral transform that can be sometime implemented with the new powerful software. We therefore conclude that the second method is much easier to implement rather than the first method. Nevertheless both methods have been proven to be efficient.

In the recent decade it was observed by several editors that many scholars using iteration method to find approximate or exact solutions of complicated equations were not investigating the stability and the convergence of their iterations. This observation led us to think that this field was much easier, and other editors said it was a child play. In order to avoid classifying this paper under this class, we will present without loss of generality the convergence of our iteration number 2 and this is done in the next section.

### 4.3. Convergence Investigation

In this section, we examine the convergence property of the approximated solution for the formation of liver zone equation. Let us consider the formation of liver zone equation in the Hilbert space *H* = *L* ^2^((*η*, *λ*) × [0, *T*]) defined as

(52) $$\begin{array}{c} H=\left\{\left(u,v\right):\left(\eta ,\lambda \right)\times \left[0,T\right],\text{with}\;\int \frac{{\left(t-\varphi \right)}^{\alpha -1}}{\Gamma \left(\alpha \right)}uvd\iota d\kappa <\infty \right\}. \end{array}$$

Then the operator is of the form

(53) $$\begin{array}{c} H\left(u,v\right)=u\left(a\left(\sigma -u-v\right)-\mu -b\overset{x}{\underset{0}{\int }}\left[cu+dv\right]d\varphi \right). \end{array}$$

That can be reduced to the following operator due to the physical properties of the problem under investigation:

(54) $$\begin{array}{c} H\left(u\right)=u\left(a\left(\sigma -u-u\right)-\mu -b\overset{x}{\underset{0}{\int }}\left[cu+du\right]d\varphi \right). \end{array}$$

The homotopy decomposition method is convergent if the following conditions are satisfied

**Hypothesis 1 .**

It is possible for us to find a positive constant, say, *F*, such that the inner product holds in the Hilbert space *H*

(55) $$\begin{array}{c} \left(H\left(u\right)-H\left(v\right),u-v\right)\geq F||u-v||,\;\forall v,u\in H. \end{array}$$

**Hypothesis 2 .**

As far as for all *v*, *u* ∈ *H* are bounded this implies, we can find a positive constant, say, *C*, such that ||*u*||, ||*v*|| ≤ *C*, then we can find Φ(*C*) > 0 such that

(56) $$\begin{array}{c} \left(H\left(u\right)-H\left(v\right),g\right)\geq \Phi \left(C\right)||u-v||||z||,\;\forall z\in H. \end{array}$$

We can therefore state the following theorem for the sufficient condition for the convergence of the generalized equation.

**Theorem 4 .**

Let us consider

(57) H(u)=∂αu∂tα=u(a(σ−u−u)−μ−b∫0x[cu+du]dφ),             with  aσ>2a+b2(c+d)

and consider the initial and boundary condition for the generalized equation; then, the homotopy decomposition method leads to a special solution of system (4).

We will present the proof of this theorem by just verifying Hypothesises 1 and 2.

**Proof**

We have that

(58) H(u)−H(v)=aσ(u−v)−2a(u2−v2) −μ(v−u)−b1u∫0x[u]dφ+b1v∫0x[v]dφ.

The above equation can be roughly converted to

(59) H(u)−H(v)=aσ(u−v)−2a(u2−v2) −μ(u−v)−b12d(u2−v2)dx=aσ(u−v)−2a(u−v)(u+v) −b12d[(u−v)(u+v)]dx.

However according to the physical property of this problem, we have that *ρ*_1_ + *ρ*_2_ the total cell density cannot exceed some fixed maximum density *σ* of cell sites, as limited by familiar phenomenon of contact inhibition; thus, we are urged to conclude that we can find a positive constant, say, *h*, such that *u* + *v* = *h*, and then,

(60) $$\begin{array}{c} =a\sigma \left(u-v\right)-2a\left(u-v\right)\left(u+v\right)-\frac{b_{1}}{2}\frac{d\left[\left(u-v\right)\left(u+v\right)\right]}{dx} \end{array}$$

can be converted to

(61) $$\begin{array}{c} =a\sigma \left(u-v\right)-2a\left(u-v\right)h-\frac{b_{1}}{2}\frac{d\left[\left(u-v\right)h\right]}{dx}. \end{array}$$

With the above in hand, we have the following inner product:

(62) (H(u)−H(v),u−v)=aσ((u−v),u−v) −2a((u−v),u−v)h −hb12d[((u−v),u−v)]dx.

According to the physical property of this problem, *u*, *v* are bounded; therefore, we can find a positive constant *F* such that (*u*, *u*), (*v*, *v*) < *F* ^2^. It follows by the use of Schwartz inequality that

(63) $$\begin{array}{c} \frac{d\left[\left(\left(u-v\right),u-v\right)\right]}{dx}\leq ||{\left(u-v\right)}_{x}||||u-v||. \end{array}$$

Now since there exists a positive constant *ω* such that ||(*u*−*v*)_*x*_|| ≤ *ω*||*u* − *v*|| then

(64) $$\begin{array}{c} \frac{d\left[\left(\left(u-v\right),u-v\right)\right]}{dx}\leq \omega {||u-v||}^{2}. \end{array}$$

On the other hand, we have that

(65) $$\begin{array}{c} \left(\left(u-v\right),u-v\right)={||u-v||}^{2}. \end{array}$$

Now making a direct substitution of (64) and (65) into (62) we arrive at

(66) $$\begin{array}{c} \left(H\left(u\right)-H\left(v\right),u-v\right)\geq F{||u-v||}^{2} \end{array}$$

with *F* = *aσ* − 2*ah* − (*hb*_1_/2). Therefore Hypothesis 1 is true for the generalized version. We will now present the proof for Hypothesis 2, as we are directly computing

(67) (H(u)−H(v),z)=aσ((u−v),z) −2a((u−v),z)h−hb12d[((u−v),z)]dx≥Φ(C)||u−v||||z||.

Therefore we can see that Hypothesis 2 is correct as well. We can conclude without fear that the homotopy decomposition method works perfectly for this generalized equation.

## 5. Conclusion

The fractional order derivative concepts have nowadays gained the world of modelling real world problem due to their properties. This has been confirmed in many studies including almost all branches of sciences. Since the formation of the liver zone is a very sensitive study issue, it has come to our mind to present the mathematical formula underpinning the formation of the liver zone within the scope of fractional derivative. We achieved this by making use of the well-known Caputo derivative because it benefits some physical properties that other including but not limited to the Rieman-Liouville and the Jumarie fractional derivative. We have with care examined the stationary solutions. Since in the recent decade several iteration methods were proposed because formal well-known analytical methods were found to be limited specially when dealing with nonlinear problem, we make use of two different techniques to get to the bottom of the generalized equation. To help readers that are not acquainted with coding theory, we presented the summary of our methods in the form of algorithms. Keeping in mind that one of the greater challenges while using iteration method is to show the convergence, we successfully proved the convergence of the second method for the generalized equation.
